# Genotypic distribution of human papillomavirus (HPV) and cervical cytology findings in 5906 Thai women undergoing cervical cancer screening programs

**DOI:** 10.1186/s13027-015-0001-5

**Published:** 2015-03-02

**Authors:** Nuttavut Kantathavorn, Chulabhorn Mahidol, Narongrit Sritana, Thaniya Sricharunrat, Natacha Phoolcharoen, Chirayu Auewarakul, Narongchai Teerayathanakul, Chantanee Taepisitpong, Siriporn Saeloo, Gaidganok Sornsamdang, Wandee Udomchaiprasertkul, Waraphorn Krongthong, Arpaporn Arnamwong

**Affiliations:** Chulabhorn Hospital, Bangkok, Thailand; Chulabhorn Research Institute, Bangkok, Thailand; Faculty of Medicine Siriraj Hospital, Mahidol University, Bangkok, Thailand; Gynecologic Oncology Unit, Chulabhorn Hospital, 54 Kamphaengphet 6 Road, Laksi, Bangkok, 10210 Thailand

**Keywords:** Cervical cancer, Cancer screening, HPV genotypes, Cervical cytology, Thailand

## Abstract

**Background:**

Cervical cancer is the major cause of morbidity and mortality in Thai women. Nevertheless, the preventive strategy such as HPV vaccination program has not been implemented at the national level. This study explored the HPV prevalence and genotypic distribution in a large cohort of Thai women.

**Methods:**

A hospital-based cervical cancer screening program at Chulabhorn Hospital, Bangkok and a population-based screening program at a rural Pathum Thani Province were conducted using liquid-based cytology and HPV genotyping.

**Results:**

Of 5906 women aged 20–70 years, Pap smear was abnormal in 4.9% and the overall HPV prevalence was 15.1%, with 6.4% high-risk (HR), 3.5% probable high-risk (PR), and 8.4% low-risk (LR) HPV. The prevalence and genotypic distribution were not significantly different between the two cohorts. Among HR-HPV genotypes, HPV52 was the most frequent (1.6%), followed by HPV16 (1.4%), HPV51 (0.9%), HPV58 (0.8%), HPV18 (0.6%), and HPV39 (0.6%). Among LR-HPV genotypes, HPV72 and HPV62 were the most frequent while HPV6 and HPV11 were rare. HPV infection was found to be proportionately high in young women, aged 20–30 years (25%) and decreasing with age (11% in women aged >50). The more severe abnormal cytology results, the higher positivity of HR-HPV infection was observed.

**Conclusions:**

In conclusion, HPV52, HPV16, and HPV51 were identified as the most common HR-HPV genotypes in Thai women. This study contributes genotypic evidence that should be essential for the development of appropriate HPV vaccination program as part of Thailand’s cervical cancer prevention strategies.

## Background

Cervical cancer is the third most common female cancer worldwide after breast cancer and colon cancer with age standardized incidence rate (ASR) of 14.0/100000 person-year [1]. Thailand is an endemic area of cervical cancer with ASR of 17.8/100000 person-year [1]. Infection with human papillomavirus (HPV) is a causal and necessary factor for the development of cervical cancer, with its prevalence in cervical cancers of 99.7% [2-6]. As a primary cervical cancer prevention, HPV vaccine has been developed to protect against HPV16 and 18 infections which are commonly found in general population and cervical cancer cases globally [7-11].

Since HPV genotypic distribution can be area-specific, it is necessary to determine its genotypic distribution before establishing health care policies and vaccination programs in each area [8,9]. This study was conducted to assess the prevalence and characteristics of HPV genotypes in Thai women as the cervical cancer prevalence has been the highest in the past many decades. Two steps were executed; Step 1 - a large hospital-based study at Chulabhorn Hospital, Bangkok followed by Step 2 - a population-based study at Bangkhayaeng District, Pathum Thani Province. The ultimate goal was to provide scientific evidence essential for the design and implementation of Thailand’s cervical cancer prevention policies.

## Materials and methods

### Study population and enrollment

After approval by the Ethical Committee for Human Research of Chulabhorn Hospital, the first hospital-based study was performed among 4550 Thai females, aged 20–70 years, who were voluntarily registered into the screening program at Chulabhorn Hospital, Bangkok, Thailand during July 19, 2011 - November 5, 2012. Exclusion criteria included absence of cervix, previous HPV vaccine vaccination, history of abnormal cytology or cervical intraepithelial neoplasia or cervical carcinoma, prior HPV infection, active disease for any types of cancer during the last 5 years, or unable to receive follow-ups throughout the program. Sixty-three women were excluded. All participants received detailed information regarding the study objectives and consented to the study. Demographics, obstetric and gynecologic history, and cervical cancer screening data were collected. The population-based study was subsequently undertaken in the Bangkhayaeng District, Pathum Thani Province using permanent living (named in the census registration) or current living status in Bangkhayaeng area at the day of pelvic examination to recruit a total of 1668 Thai females, aged 20–70 years who then underwent cervical cancer screening during February 4-June 16, 2013. Exclusion criteria were the same as our previous study.

### Sample collection and preparation

Samples were obtained using a cytobrush by gynecologic oncologists or well-trained general practitioners for pelvic examination of Chulabhorn Hospital. The brush was then placed in the preservative fluid in the BD SurePath Pap test kit (BD Diagnostics-Tripath, Burlington, NC, USA) for both liquid-based cytology and HPV DNA testing. The investigators performing HPV typing were blinded to cytology results. All cervical cytology slides were interpreted per normal routine by qualified pathologists at Chulabhorn Hospital, using the Bethesda 2001 report system [12].

### HPV genotyping

For the identification of HPV genotypes, we used the Linear array HPV testing (Roche, USA). This kit was capable of identifying 37 HPV types including 12 high-risk (HR), 8 probable high-risk (PR), and 17 low-risk (LR) types those classified by oncogenic potentiality [13-16]. In brief, 450-bp fragments from the L1 region of the virus were first amplified by polymerase chain reaction (PCR) of target DNA, followed by hybridization using a reverse line blot system for simultaneous detection of up to 37 HPV genotypes (i.e., genotypes 6, 11, 16, 18, 26, 31, 33, 35, 39, 40, 42, 45, 51, 52, 53, 54, 55, 56, 58, 59, 61, 62, 64, 66, 67, 68, 69, 70, 71, 72, 73, 81, 82, 83, 84, IS39, and CP6108).

### PCR amplification

Each 100 μL reaction consisted of 50 μL working master mix and 50 μL of DNA sample. Amplification was performed in an Applied Biosystems GeneAmp PCR System 9700 using the recommended parameters: 50°C for 2 min, 95°C for 9 min and 40 cycles of 95°C for 30 s, 55°C for 1 min, 72°C for 1 min and 72°C for 5 min before holding indefinitely at 72°C.

### Hybridization to the oligonucleotide probe

Following PCR amplification, the HPV and the ß-globin amplicon were chemically denatured to form single stranded DNA by addition of 100 μL Denaturation Solution. Aliquots 100 μL of denatured amplicon were then transferred to the appropriate well of typing tray containing hybridization buffer and single LINEAR ARRAY HPV Genotyping Strip coated with HPV and ß-globin probe lines. The biotin-labeled amplicon was hybridized to the oligonucleotide probes only if the amplicon contained the matching sequence of complementary probe.

### Colorimetric determination

A blue colored complex precipitated at the probe positions where hybridization occurred. Then, the linear array HPV genotyping strip was read visually by comparing the pattern of blue lines to the linear array HPV genotyping test reference guide.

### Statistical analysis

Descriptive statistics were used to determine the distribution types and frequencies of HPV positivity, age, and cytology results. Frequency tables were done for qualitative variables. Independent samples t-test was used compare the means of age and Pearson’s chi-square test were performed to verify the association of demographic data and HPV prevalence between the two cohorts. p <0.05 was considered statistically significant. Licensed Stata program version 12 was used for analysis.

## Results

### Demographic characteristics of 5906 Thai women

Demographic characteristics of 2 cervical cancer screening populations were shown in Table 1. Of 5906 women, the median age was 45 years with a range of 20–70 years. Approximately two-thirds of them were pre-menopausal and had prior pregnancy history. The proportion of those having children and being married was higher in the Bangkhayaeng cohort than Chulabhorn Hospital cohort. Women in the Bangkhayaeng District were of lower educational level as compared to the Chulabhorn Hospital cohort.

**Table 1:** **Demographic characteristics of Thai women in two study cohorts**

**Demographics**	**Total (N =5906)**	**Chulabhorn hospital (N = 4487)**	**Bangkhayaeng district (N = 1419)**	**p-value**
Age				
Mean (years)	44.8	44.8	44.8	0.986^1^
Median (years)	45.0	45.0	45.0	
Range (years)	20-70	20-70	20-70	
20-30 years (%)	634 (10.7)	481 (10.7)	153 (10.8)	
31-70 years (%)	5272 (89.3)	4006 (89.3)	1266 (89.2)	
Parity^†^				<0.001^2,^*
Nulliparous	1867 (31.7)	1658 (36.9)	209 (14.9)	
Multiparous	4019 (68.3)	2829 (63.1)	1190 (85.1)	
Menopause^†^				0.714^2^
Pre-menopause	4012 (68.2)	3064 (68.3)	948 (67.8)	
Post-menopause	1874 (31.8)	1423 (31.7)	451 (32.2)	
Marital status				<0.001^2,^*
Single	1448 (24.5)	1319 (29.4)	129 (9.1)	
Married	3619 (61.3)	2530 (56.4)	1089 (76.7)	
Divorced	839 (14.2)	638 (14.2)	201 (14.2)	
Number of life-time sex partner(s)				
0 (virgin)	-	430 (9.6)	-	
1	-	2827 (63.0)	-	
1+	-	1230 (27.4)	-	
Contraception use^†^				<0.001^2,^*
Yes	3002 (51.0)	2002 (44.6)	1000 (71.5)	
No	2884 (49.0)	2485 (55.4)	399 (28.5)	
Education^†^				<0.001^2,^*
No education	56 (1.0)	27 (0.6)	29 (2.1)	
Primary education	1319 (22.4)	678 (15.1)	641 (45.8)	
High school	1025 (17.4)	651 (14.5)	374 (26.7)	
Vocation school	820 (13.9)	650 (14.5)	170 (12.2)	
Bachelor degree	2162 (36.7)	1991 (44.4)	171 (12.2)	
Postgraduate	504 (8.6)	490 (10.9)	14 (1.0)	

^†^missing data for Bangkayaeng Cohort 20 cases (1.4%).

^1^Independent samples t-test.

^2^Pearson’s chi-square test.

*p < 0.05.

### HPV prevalence and genotypic distribution

**Figure 1:**
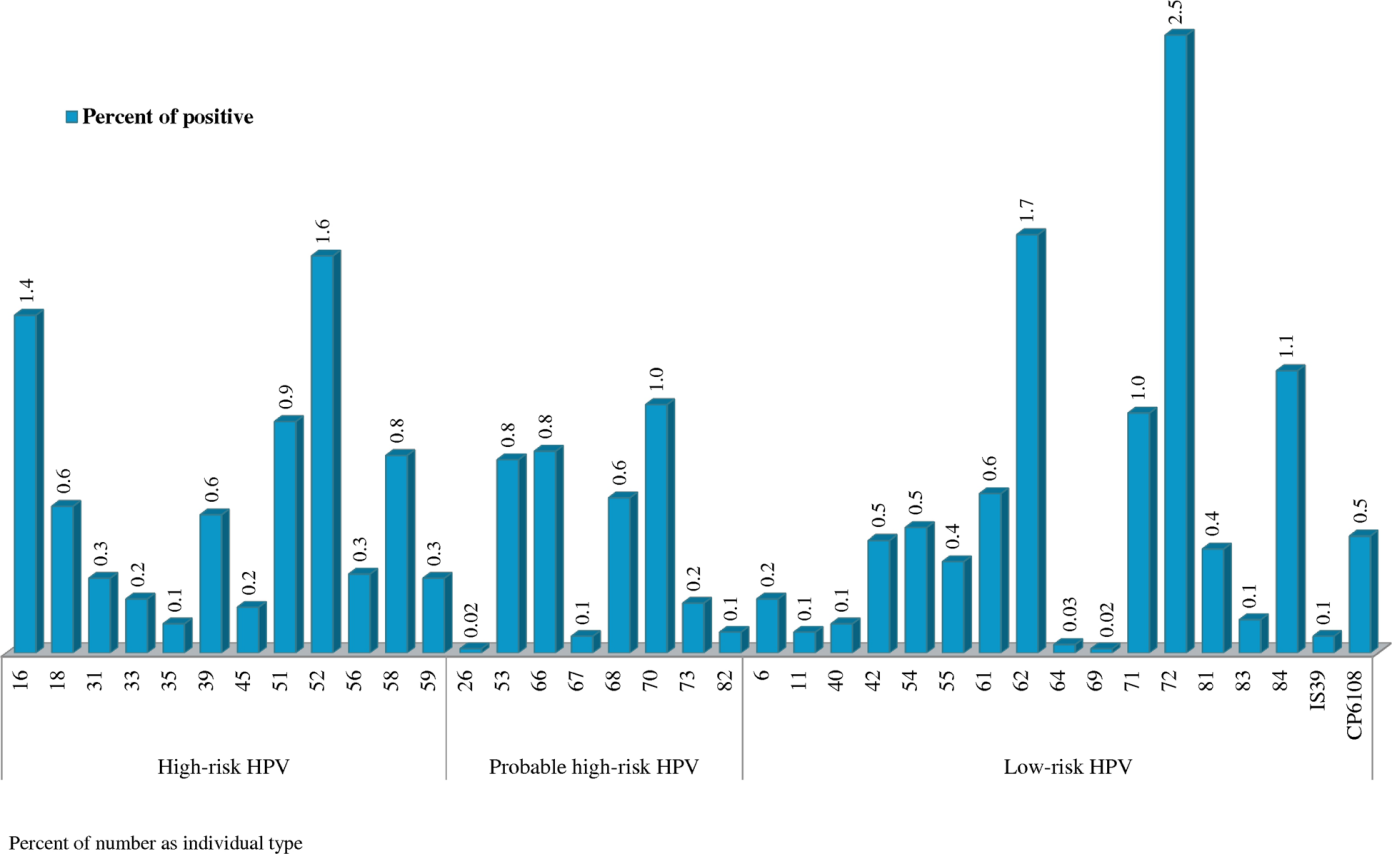
**Overall HPV genotyping in 5906 Thai females.**

Table 2 demonstrates the overall HPV prevalence among 5906 Thai women was 15.1%, with 6.4% high risk (HR), 3.5% probable high risk (PR), and 8.4% low risk (LR) HPV. No significant difference was identified on the HPV prevalence between the two cohorts (p = 0.178). Overall, the most frequent HR-HPV types consisted of HPV52 (1.6%), HPV16 (1.4%), HPV51 (0.9%), HPV58 (0.8%), HPV18 (0.6%), and HPV39 (0.6%). Common PR-HPV types were HPV70 (1.0%), HPV66 (0.8%), HPV53 (0.8%), and HPV68 (0.6%). LR-HPV subtypes were HPV72 (2.5%), HPV62 (1.7%), HPV84 (1.1%), HPV71 (1.0%), and HPV61 (0.6%) (Figure 1).

**Table 2:** **Comparison of the HPV prevalence in Thai women enrolled at Chulabhorn hospital or Bangkhayaeng district**

	**Total**	**Chulabhorn hospital**	**Bangkhayaeng district**	**p-value**
**Period (year)**		2011-2012	2013	
**Total**	5906	4487	1419	
**HPV**				0.178
**Negative**	5016 (84.9)	3795 (84.6)	1221 (86.0)	
**Positive**	890 (15.1)	692 (15.4)	198 (14.0)	
**High risk HPV** ^**†**^	376 (6.4)	292 (6.5)	84 (5.9)	0.429
**Probable HR HPV** ^**†**^	204 (3.5)	156 (3.5)	48 (3.4)	0.866
**Low risk HPV** ^**†**^	495 (8.4)	394 (8.8)	101 (7.1)	0.049

^†^Number (%), as individual type.

**Figure 2:**
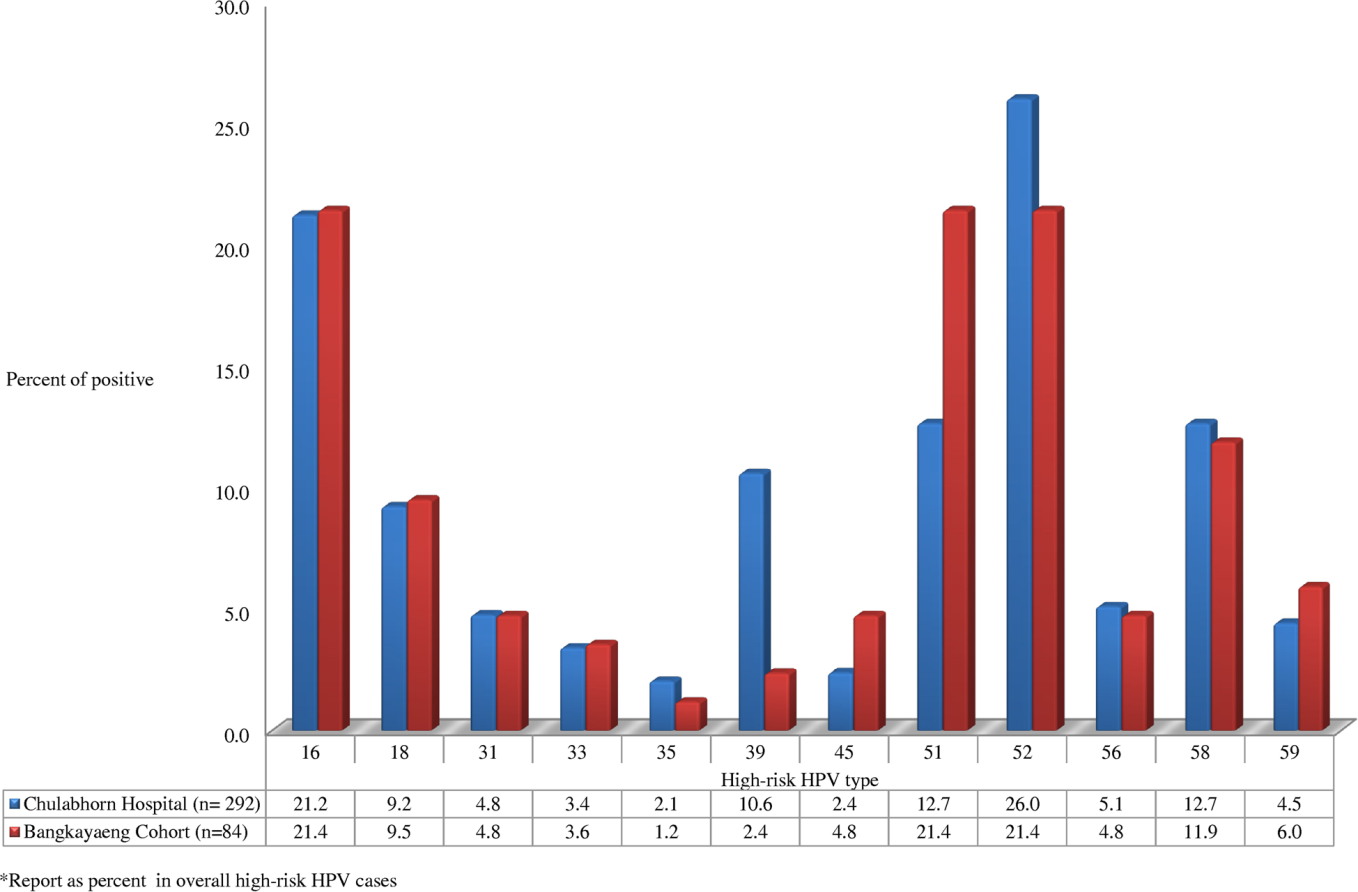
**HR-HPV prevalence in 2 cohorts.**

Figure 2 shows the distribution of HR-HPV prevalence in each cohort. The most common HR-HPV in Chulabhorn Hospital Cohort was HPV52, 16, 51, 58, 39 and 18 (26%, 21.2%, 12.7%, 12.7%, 10.6%, and 9.2%, respectively). The most common HR-HPV in Bangkhayaeng District Cohort was HPV16, 51, 52, 58, 18 and 59 (21.4%, 21.4%, 21.4%, 12.7%, 9.5%, and 6.2%, respectively).

### Age-specific prevalence of HPV infection and abnormal Pap smear

Pap smear was abnormal in 4.9% of the population. The abnormal Pap smear rate was highest in those young women aged 20–30 years (6.6%) and decreased by advancing age (Table 3). In addition, HPV infection was found most frequent among women aged < 30 years, with the prevalence of 24.8% (13.2% of HR-HPV), and decreased by increasing age.

**Table 3:** **HPV prevalence and risk groups among 5906 women classified by age range and cytology results**

**HPV Type**	**Age range (year)**						**Normal cytology**	**Abnormal cytology**
	**20-30**	**31-40**	**41-50**	**51-60**	**61-70**	**Total**		
**N**	634	1479	1936	1368	489	5906	5614 (95.1)	292 (4.9)
**Pap smear results**								
**Normal**	592 (93.4)	1403 (94.9)	1835 (94.8)	1312 (95.9)	472 (96.5)	5614 (95.1)	-	-
**Abnormal**	42 (6.6)	76 (5.1)	101 (5.2)	56 (4.1)	17 (3.5)	292 (4.9)	-	-
**HPV results**								
**HPV negative**	477 (75.2)	1221 (82.6)	1666 (86.1)	1217 (89.0)	435 (89.0)	5016 (84.9)	4866 (86.7)	150 (51.4)
**HPV positive**	157 (24.8)	258 (17.4)	270 (13.9)	151 (11.0)	54 (11.0)	890 (15.1)	748 (13.3)	142 (48.6)
**High risk HPV**	84 (13.2)	126 (8.5)	101 (5.2)	50 (3.7)	15 (3.1)	376 (6.4)	285 (5.1)	91 (31.2)
**Probable HR**	43 (6.8)	64 (4.3)	61 (3.2)	26 (1.9)	10 (2.0)	204 (3.5)	169 (3.0)	35 (12.0)
**Low risk HPV**	76 (12.0)	124 (8.4)	157 (8.1)	103 (7.5)	35 (7.2)	495 (8.4)	431 (7.7)	64 (21.9)

•HR: High risk, PR: probable HR, LR: low risk.

•Values given as number (percentage) as individual type.

### HPV infection frequency and abnormal cervical cytology

**Figure 3:**
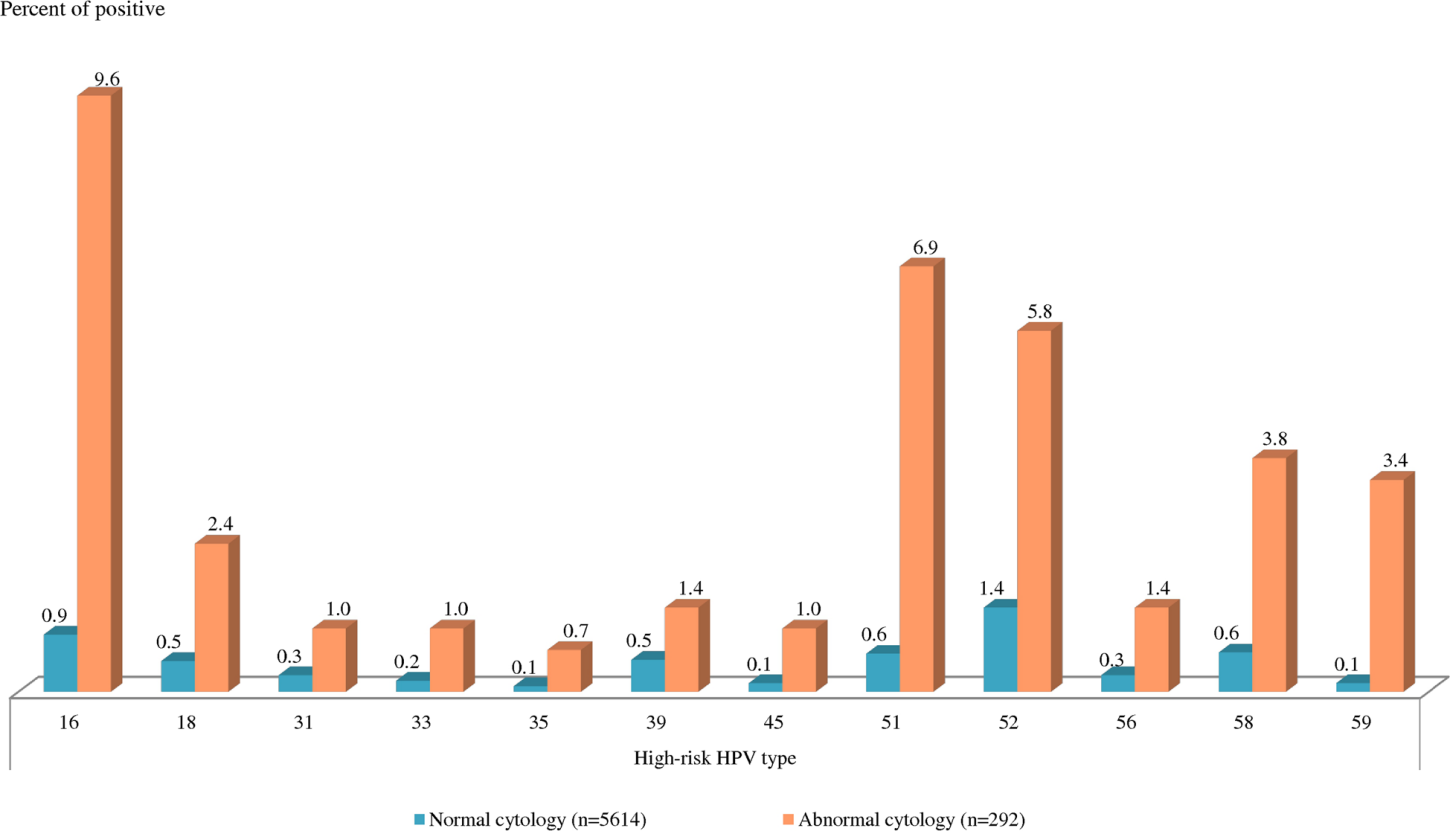
**HR-HPV genotyping in normal and abnormal cytology.**

The HPV positivity rates in cases with atypical squamous cells of undetermined significance (ASC-US) (n = 189, 3.2%), atypical squamous cells, cannot exclude high-grade squamous intraepithelial lesion (ASC-H) (n = 11, 0.2%), low grade squamous intraepithelial lesion (LSIL) (n = 66, 1.1%), and high grade squamous intraepithelial lesion (HSIL) (n = 20, 0.3%) was 31.2%, 45.5%, 86.4% and 90.0%, respectively. The more severe cytological result, the higher HPV-positive proportion was found.HPV, HR-HPV positive samples with normal cytology in 5614 women amounted to 13.3%, 5.1%, respectively. The most common HR-HPV type was HPV52 (1.4%), HPV16 (0.9%), HPV51 (0.6%), HPV58 (0.6%), HPV18 (0.5%), and HPV39 (0.5%), respectively. The overall HPV prevalence in 292 women with abnormal cytology was 48.6% (HR-HPV 31.2%).The most common HR-HPV types in this group included HPV16 (9.6%), HPV51 (6.9%), HPV52 (5.8%), HPV58 (3.8%), HPV59 (3.4%), and HPV18 (2.4%) as shown in Figure 3.

## Discussion

We investigated the prevalence and distribution of HPV genotypes among 5906 Thai women enrolled for cervical cancer screening at Chulabhorn Hospital, Bangkok, or Bangkhayaeng District, Pathum Thani and revealed the 15% HPV infection rate among these women. The overall prevalence in both hospital-based and population-based cohorts was not statistically different despite the significant differences in the cohorts’ demographic characteristics as shown in Table 1. The rural Bangkhayaeng District appeared to comprise more uneducated, multiparous, and married women than the Chulabhorn Hospital-based cohort in Bangkok. The genotypic data was not different between the two cohorts suggesting that the rate and type of HPV infections in Thai women in this study were comparable regardless of their geographic locations and education levels. However, larger studies involving more sub-regions of Thailand may be needed.

Major studies from the Western countries reported the prevalence of HR-HPV infections around 11.3%-18.3% which were higher than our results [17-21]. Disparate data from three previous studies conducted in Thailand currently exist showed the lower prevalence of HPV infections (6.3%-8.7%) which could be attributed to different genotypic methods utilized in each study [22-24]. A study by the National Cancer Institute of Thailand reported the overall prevalence of 13 HPV genotypes (16, 18, 31, 33, 35, 39, 45, 51, 52, 56, 58, 59, 68) of 8.2% by Hybrid Capture 2 hybridization assay method without specific genotypic data [22]. Another Thai study utilizing polymerase chain reaction (PCR) for E1 amplification for 20 HPV genotypes (HR type 16, 18, 30, 31, 33, 35, 39, 42, 45, 51, 52, 56, 58, 59, 68, 73, 82, PR type 66, and LR type 6, 11) found the HPV prevalence of 8.7% among 1662 women in the screening setting [23]. The higher HPV prevalence in our cohort that differs from other Thai cohorts may reflect our more comprehensive genotypic analysis that was aimed to detect more HPV genotypes (37 genotypes) than other Thai studies, regardless of cervical cancer risk categories. Nevertheless, the HR-HPV prevalence in this study of 6.4% was comparable to most previously reported Thai studies [22-24].

With respect to the HR-HPV genotypes that are the major risk factors for cervical cancer, it was of interest to find that in our Chulabhorn Hospital Cohort, the most common genotype was HPV52 followed by HPV16 and HPV51. This finding was in contrast to most studies in the Western countries and two smaller studies in Thailand in which HPV16 was identified as the most frequent [23,24]. Nevertheless, HPV16 and HPV51 were also well represented together with HPV52 as the top HR-HPV genotypes in our Bangkhayaeng District Cohort. When classified by continents, it was observed that HPV16 was the first top-ranking HPV genotype as shown in Table 4, whereas the second and other subsequent rankings were distinctively changed according to each region [9]. For instance, HPV52 and HPV31 were the second top-rankings in Africa and Europe, respectively. Meanwhile, in the Asia continent, the most commonly found HPV included HPV16, HPV18, and HPV52, with the prevalence of 2.5%, 1.4% and 0.7%, respectively [25-32]. Parkin et al. studied the HPV prevalence in a normal cytology group of 28998 Eastern Asian female cases and revealed that the most common HR-HPV types were HPV16, HPV52, HPV58, HPV18, HPV56, and HPV51, with the prevalence of 2.7%, 1.3%, 1.2%, 0.7%, 0.7%, 0.7%, respectively [27]. A study in China also showed a high frequency of HPV16, HPV52, HPV58, and HPV18 [26]. Bruni et al. performed meta-analysis on HPV prevalence in one million normal cytology woman which showed HR-HPV infection rate of 11.7% and the five most common HR-HPV types worldwide were HPV16 (3.2%), HPV18 (1.4%), HPV52 (0.9%), HPV31 (0.8%), and HPV58 (0.7%) [9]. Recently, Zhao et al. reported that the most commonly HR-HPV in cervical specimens at baseline from Chinese women aged 18–25 years were HPV-52 (4.0%) and HPV-16 (3.7%) [33]. Interestingly, HPV18, the most frequently detected HR-HPV after HPV16 worldwide, was surprisingly uncommon in our study population. Our data is consistent with results from previous Thai studies that reported a very low prevalence of HPV18 [23,24].

**Table 4:** **Comparison of HR-HPV prevalence and genotypic distribution in screening settings in various countries**

**Study/year**	**Site/N**	**HPV Test**	HR-HPV*	**16**	**18**	**31**	**33**	**35**	**39**	**45**	**51**	**52**	**56**	**58**	**59**
			**N (%)**												
Ralston Howe E (15)	USA	PCR	13444	4507	881	1757	524	479	306	734	26	2009	437	865	919
2009	73371		(18.3)	(33.5)	(6.6)	(13.1)	(3.9)	(3.6)	(2.3)	(5.5)	(0.2)	(14.9)	(3.3)	(6.4)	(6.8)
Dickson EL (16) **	USA	PCR	11.9%	4.1%	0.7%	1.9%	0.4%	0.3%		0.5%	-	2.1%	-	1.1%	0.8%
2013	309471		(100)	(34.5)	(5.9)	(16.0)	(3.4)	(2.5)	-	(4.2)		(17.6)		(9.2)	(6.7)
Agarossi A (17)	Italy	HC2 followed by PCR	1403	415	150	211	61	67	111	68	132	67	142	120	45
2009	9946		(14.1)	(29.6)	(10.7)	(15.0)	(4.3)	(4.8)	(7.9)	(4.8)	(9.4)	(4.8)	(10.1)	(8.6)	(3.2)
Anderson L (18) **	Ireland	Cobas4800 test followed by Linear array	16.6%	3.2%	1.2%	1.8%	1.2%	0.5%	1.2%	1.0%	1.6%	1.5%	1.0%	0.8%	1.6%
2012	5712		(100)	(19.3)	(7.2)	(10.8)	(7.2)	(3.0)	(7.2)	(6.0)	(9.6)	(9.0)	(6.0)	(4.8)	(9.6)
Ucakar V (19)	Slovenia	HC2/Real Time PCR followed by Linear array	502	155	46	114	32	9	50	42	81	78	31	29	29
2012	4431		(11.3)	(30.9)	(9.2)	(22.7)	(6.4)	(1.8)	(10.0)	(8.4)	(16.1)	(15.5)	(6.2)	(5.8)	(5.8)
INOUE M (23)	Japan	HC2 followed by HPVDNAChip (Biomedlab Co., South Korea)	632	167	73	50	17	7	43	20	98	189	100	131	39
2006	8156		(7.7)	(22.7)	(9.9)	(6.8)	(2.3)	(1.0)	(5.9)	(2.7)	(13.3)	(25.7)	(13.6)	(17.8)	(5.3)
Li H (24)	China	HPV GenoArray (Hybribio, Hong Kong)	755	233	64	26	30	3	44	9	20	163	27	119	17
2013	3640		(20.7)	(30.9)	(8.5)	(3.4)	(4.0)	(0.4)	(5.8)	(1.2)	(2.6)	(21.5)	(3.6)	(15.8)	(2.3)
Zhoa FH (31)	China	SPF PCR-DEIA-LiPA (LaboBiomedial Product, Rijswijk, the Netherlands)	924	222	74	59	65	31	81	33	102	243	69	90	22
2014	6035		(15.3)	(3.7)	(1.2)	(1.0)	(1.1)	(0.5)	(1.3)	(0.5)	(1.7)	(4.0)	(1.1)	(1.5)	(0.4)
This study	Thailand	Linear array (Roche, USA)	376	80	35	18	13	7	33	11	55	94	19	47	18
2015	5906														
			(6.4)	(21.2)	(9.3)	(4.8)	(3.5)	(1.9)	(8.8)	(2.9)	(14.6)	(25.0)	(5.1)	(12.5)	(4.8)

*HR-HPV included 12 HPV types as 16, 18, 31, 33, 35, 39, 45, 51, 52, 56, 58, and 59.

**Reported as percentage in total population.

HC2: Hybrid Capture 2 (Qiagen, Hilden, Germany), PCR: polymerase chain reaction.

When considering the significance of HPV52 in Thai women compared to worldwide invasive cancer cases in the meta-analysis of 30848 cases from 243 studies, the prevalence of global HR-HPV was 89.9%, with HPV16 and HPV18 as the most common types, followed by HPV types 58, 33, 45, 31, 52, 35, 59, 36 and 51, respectively [11]. In our study of 292 women with abnormal cytology, about half of them had HPV infections and the most common HR-HPV types continued to be the common HR-HPV types found in the normal cytology cases, including HPV16, HPV51, HPV52 followed by HPV58, HPV59, and HPV18, respectively. HPV52 was also frequently identified in two small cervical cancer studies reported from Thailand [34,35]. A study by Thai NCI in 155 cases of cervical cancer showed that the most commonly identified types were HPV16, HPV18, HPV52, HPV58, and HPV33 [35]. Similar results from a study by Chiang Mai University Hospital in the Northern Thailand in 99 cases of cervical cancer showed that the most frequently found HR-HPV consisted of HPV16, HPV52, HPV18, HPV33, and HPV58 [34]. HPV52 thus plays an important role in the Thai population.

HPV prevalence is strongly associated with age worldwide [8,9]. We noted a decline in the HPV prevalence, both HR-HPV and non-HR-HPV genotypes, with increasing age of the women in this study. It is also of interest to find a lower rate of HPV infection (24.8%) and HR-HPV genotypes (13.2%) in our young population aged <30 years as compared to worldwide or Western continent data, which showed a higher HR-HPV prevalence of 20-30% in women aged <30 years [8,9]. A follow-up study of these young women is presently ongoing to determine the natural history of HPV infections in this particular subgroup. Moreover, 13.3% of women in our study had HPV infection despite negative cytology results. This data was comparable to a meta-analysis performed by de Sanjose et al. which showed HR-HPV prevalence of 10.4% in women with negative cytology results [8]. These women infected with HPV despite negative cytology results are also being followed to assess the persistence of HPV and CIN development.

Future cervical cancer screening is moving towards HPV-based screening because of its very high sensitivity [36-38]. A randomized control study by Mayrand et al. in 10154 women showed that HPV testing alone had a higher sensitivity (94.6%) for detection of CIN2+ when compared to conventional Pap smears (55.4%) [36]. In a country with a low prevalence of HR-HPV as Thailand, primary HR-HPV testing would have a high yield because the positive rate is not too high and will not lead to overwhelming referral for secondary triage or even colposcopy. Pap smear of unnecessary cases could be possibly reduced by 93.6%.

In conclusion, this study represents the largest report from Thailand with respect to detailed and comprehensive genotypic analysis of HPV subtypes. The prevalence and genotypic distribution of HPV did not significantly differ between hospital-based and population-based cohorts. HPV52 was the most frequently identified high-risk genotype in Thai women followed by HPV16 and HPV51. The majority of Thai women undergoing cervical cancer screening programs were not infected by readily available vaccine-preventable HPV genotypes (HPV16, HPV18, HPV6, and HPV11). This study thus provides scientific evidence essential for the development of appropriate HPV vaccination programs targeted for certain HR-HPV genotypes prevalent in the Thai women.

## Abbreviations

ASC-H: Atypical squamous cells, cannot exclude high-grade squamous intraepithelial lesion
ASC-US: Atypical squamous cells of undetermined significance
ASR: Age standardized incidence rate
CIN: Cervical intraepithelial neoplasia (CIN)
HPV: Human papillomavirus
HR: High risk
HSIL: High grade squamous intraepithelial lesion
LR: Low risk
LSIL: Low grade squamous intraepithelial lesion
NCI: National Cancer Institute
PCR: Polymerase chain reaction
PR: Probable high risk
